# Bivalent DNA vaccine induces significant immune responses against infectious hematopoietic necrosis virus and infectious pancreatic necrosis virus in rainbow trout

**DOI:** 10.1038/s41598-017-06143-w

**Published:** 2017-07-18

**Authors:** Liming Xu, Jingzhuang Zhao, Miao Liu, Guangming Ren, Feng Jian, Jiasheng Yin, Ji Feng, Hongbai Liu, Tongyan Lu

**Affiliations:** 1 Heilongjiang River Fishery Research Institute, Chinese Academy of Fishery Sciences, Harbin, 150070 P.R. China; 2 Benxi AgriMarine Industries Inc., Benxi, 117000 P.R. China

## Abstract

Infectious hematopoietic necrosis virus (IHNV) and infectious pancreatic necrosis virus (IPNV) are important pathogens of salmon and trout. An active bivalent DNA vaccine was constructed with the glycoprotein gene of Chinese IHNV isolate Sn1203 and VP2–VP3 gene of Chinese IPNV isolate ChRtm213. Rainbow trout (5 g) were vaccinated by intramuscular injection with 1.0 µg of the bivalent DNA vaccine and then challenged with an intraperitoneal injection of IHNV, IPNV, or both, at 30 and 60 days post-vaccination (d.p.v.). High protection rates against IHNV were observed, with 6% and 10% cumulative mortality, respectively, compared with 90–94% in the mock-vaccinated groups. IPNV loads (531-fold and 135-fold, respectively) were significantly reduced in the anterior kidneys of the vaccinated trout. Significant protection against co-infection with IHNV and IPNV was observed, with cumulative mortality rates of 6.67% and 3.33%, respectively, compared with 50.0% and 43.3%, respectively, in the mock-vaccinated groups. No detectable infective IHNV or IPNV was recovered from vaccinated trout co-infected with IHNV and IPNV. The bivalent DNA vaccine increased the expression of Mx-1 and IFN-γ at 4, 7, and 15 d.p.v, and IgM at 21 d.p.v., and induced high titres (≥160) of IHNV and IPNV neutralizing antibodies at 30 and 60 d.p.v.

## Introduction

Infectious hematopoietic necrosis virus (IHNV) and infectious pancreatic necrosis virus (IPNV) are the causative agents of infectious hematopoietic necrosis (IHN) and infectious pancreatic necrosis (IPN), respectively. IHNV is an enveloped non-segmented single-stranded negative RNA virus in the genus *Novirhabdovirus* within the family *Rhabdoviridae*, which is responsible for major losses in salmonid production. The IHNV genome contains six genes in the order 3′-N–P–M–G–NV–L-5′, encoding the nucleocapsid protein (N), phosphoprotein (P), matrix protein (M), glycoprotein (G), non-virion protein (NV), and polymerase protein (L), respectively^1^. The virus infects several salmonid species^2^, with mortality rates of 80–90%. IPNV belongs to the family *Birnaviridae* and has a bisegmented genome of double-stranded RNA (segments A and B). Segment A encodes VP2 and VP3, the two major structural proteins of the virus. VP2 contains the determinants of antigenicity and virulence, and major neutralizing epitopes, and is important for IPNV immunogenicity^3^. VP3 is an internal structural protein in which some neutralizing epitopes have been identified^4^. Segment B contain a single open reading frame encoding VP1.

IHNV and IPNV are widespread in salmonid hatcheries from the Americas to Europe, Asia, and Australia^5, 6^. Fish that survive an IHNV or IPNV infection may become carriers of the virus for long periods and consequently transmit the virus to other susceptible fish or shellfish species^7–9^. Vaccination is one of the best methods for controlling these diseases. Various candidate IHNV vaccines have been designed, including attenuated vaccines^10, 11^, killed virus^12^, and DNA vaccines^2, 13^. Although the IHN DNA vaccine provided almost full protection to rainbow trout against IHNV infection, only one DNA vaccine has been commercialized, by the Canadian Food Inspection Agency^14^. Different kinds of IPNV vaccines have been reported for fish, including inactivated vaccines^15^, attenuated vaccines^16^, DNA vaccines^17–22^, and subunit vaccines^23–27^, but protection is not always complete^5, 17, 28^.

Although vaccines against IHNV and IPNV have been commercialized in several countries, outbreaks of IPNV and IHNV are still a major problem in modern aquaculture around the world. This may be because fish in the field can be exposed to several pathogens simultaneously. Therefore, multivalent vaccines against two or more pathogens are valuable tools in aquaculture^29^. Previous studies have demonstrated the co-infection of rainbow trout with IHNV and IPNV under natural conditions^30, 31^. Therefore, in this study, a bivalent DNA vaccine was constructed with the G gene of Chinese IHNV isolate Sn1203^32^ and the VP2–VP3 genes of Chinese IPNV isolate ChRtm213^33^. Here, we report the successful design and construction of this bivalent DNA vaccine, designated pCh-IHN/IPN, which induced protective immune responses against IHNV infection, IPNV infection, and co-infection with IHNV and IPNV in the rainbow trout. This is the first study to construct a bivalent DNA vaccine targeting diverse viral pathogens in salmon and trout. This may be a feasible strategy for controlling IHN and IPN worldwide.

## Results

### Expression of antigen genes

**Figure 1:**
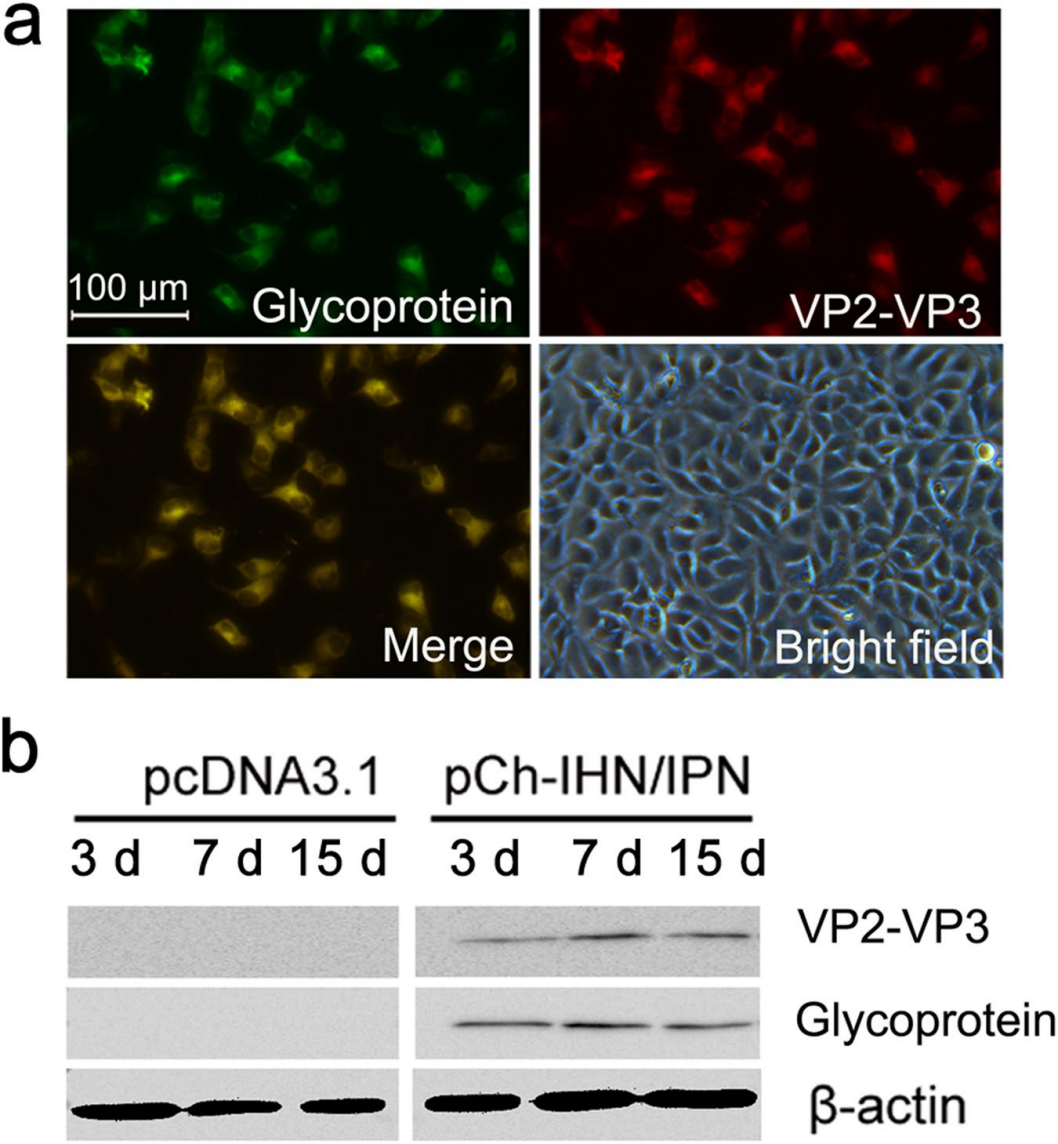
*In vitro* and *in vivo* expression of both antigen genes from the pCh-IHN/IPN DNA vaccine. An immunofluorescence antibody test confirmed the expression of both antigen genes *in vitro* (**a**). EPC cells transfected with pCh-IHN/IPN were incubated with an IHNV-glycoprotein-specific rabbit polyclonal antibody and a Cy3-conjugated goat anti-rabbit-IgG secondary antibody or a mouse anti-IPNV-VP2 polyclonal antibody and a fluorescein isothiocyanate (FITC)-conjugated goat anti-mouse-IgG antibody. EPC cells transfected with the pcDNA3.1 vector and treated identically were used as the negative control. Western blotting of muscle samples from vaccinated rainbow trout (n = 5), collected at 3, 7, and 15 days post-vaccination, detected the expression of both antigen genes *in vivo* (**b**). Muscle samples from pcDNA3.1-mock-vaccinated trout were used as the negative controls. β-Actin was used as the reference protein.

Epithelioma papulosum cyprini (EPC) cells were transfected with the bivalent DNA vaccine pCh-IHN/IPN with a routine procedure (see Supplementary Figure S1 for a map of the bivalent DNA vaccine). The expression of both antigen genes was confirmed *in vitro* and *in vivo* with an immunofluorescence antibody test (IFAT) and western blotting, respectively. In the IFAT, specific green and red fluorescence was observed simultaneously in the same cells, which had been successfully transfected with pCh-IHN/IPN. Specific yellow fluorescence was observed in the merged images, whereas no specific fluorescent signal was observed in cells transfected with pcDNA3.1 (Fig. 1a). On a western blot, clear and specific bands were observed at 3, 7, and 15 days post-vaccination (d.p.v.) in muscle tissues from the sites of vaccine delivery in rainbow trout immunized with pCh-IHN/IPN, whereas no bands were observed in the lanes containing muscle tissues from empty-vector-immunized rainbow trout. The reference β-actin protein was observed in each lane (Fig. 1b) (full-length gels with markers are shown in Supplementary Figure S2). These results indicate that the G and VP2–VP3 genes were efficiently expressed by the pCh-IHN/IPN DNA vaccine in fish cells.

### Protection against IHNV afforded by the DNA vaccine

**Figure 2:**
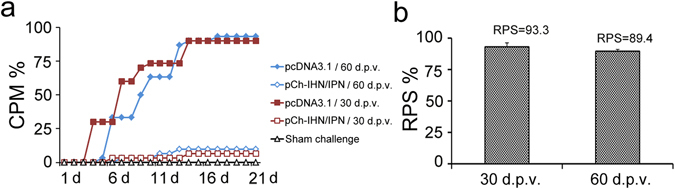
Cumulative percentage mortality (CPM) curves for pCh-IHN/IPN-vaccinated rainbow trout challenged with IHNV strain Sn1203 at 30 or 60 d.p.v. Rainbow trout injected with plasmid pcDNA3.1 (vector) were used as the negative controls. Duplicate groups of 30 fish were challenged with an intraperitoneal injection of 10^2^ plaque-forming units of IHNV Sn-1203 per fish. No mortality was observed in the sham-infected control group, and no significant differences were observed in mortality between any replicates within any treatment group.

Replicate groups of 50 rainbow trout fry (5 g), intramuscularly (i.m.) injected with 1.0 µg of pCh-IHN/IPN or treated with the various controls, were challenged at 30 or 60 d.p.v. The rainbow trout were significantly protected at 30 and 60 days compared with the pcDNA3.1-mock-vaccinated group (P < 0.05), and showed 6–10% cumulative mortality compared with 90–94% cumulative mortality in the pcDNA3.1-mock-vaccinated group. No mortality was observed in the sham-infected group (Fig. 2a), and no significant difference in mortality was observed between any replicates within any of the treatment groups in either experiment (data not shown). There was no significant difference in the relative percentage survival (RPS) at 30 and 60 d.p.v. (Fig. 2b; P > 0.05).

### IPNV load

**Figure 3:**
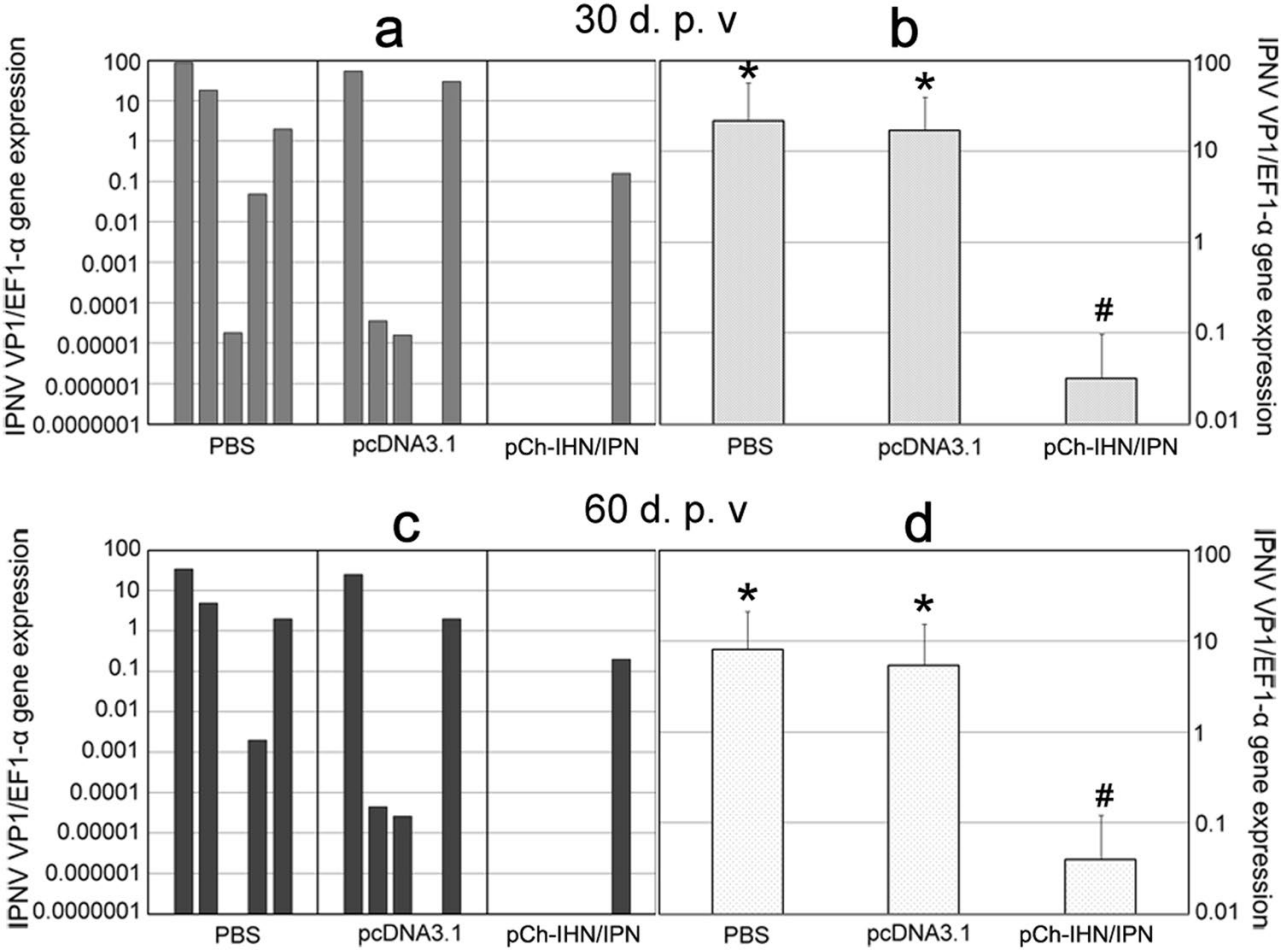
Quantitative reverse transcription–PCR determination of IPNV load using VP1 gene expression in the anterior kidneys of vaccinated rainbow trout (n = 5) challenged with IPNV strain ChRtm213 at 30 or 60 d.p.v. IPNV loads were measured at 15 days post challenge. Rainbow trout injected with plasmid pcDNA3.1 alone or with PBS were used as the negative controls. EF-α was used to normalize the expression of the IPNV VP1 gene. Individual VP1 gene expression levels (**a**,**c**) and average expression levels (**b**,**d**) are shown separately. Differences were analysed, and different symbols above the bars indicate significant differences (P < 0.05).

Duplicate groups of 50 rainbow trout that were vaccinated i.m. with 1.0 µg of pCh-IHN/IPN or treated with the various controls were challenged with an intraperitoneal (i.p.) injection of IPNV ChRtm213 at 30 and 60 d.p.v. We evaluated the protection afforded by the bivalent DNA vaccine against IPNV by measuring the viral load in the anterior kidney in terms of VP1 gene expression 15 days after challenge (Fig. 3). The fold changes in the viral load in the bivalent-DNA-vaccine-treated group were calculated relative to those in the pcDNA3.1-mock-vaccinated group. The EF-α gene was used to normalize VP1 gene expression, and the individual VP1 gene expression levels and average expression levels were described separately. The levels of virus varied greatly in the five phosphate-buffered saline (PBS)-injected fish and empty-vector-injected fish (Fig. 3a,b). The average fold changes in the viral load in fish challenged at 30 and 60 d.p.v. were 531-fold and 135-fold, respectively, and IPNV was only detected in one of the five fish sampled in both cases. These results indicate that the average viral load was significantly reduced in fish vaccinated with pCh-IHN/IPN compared with that in the empty-vector-treated group (Fig. 3b,d; P < 0.05).

### Protection conferred by the vaccine against co-infection

**Figure 4:**
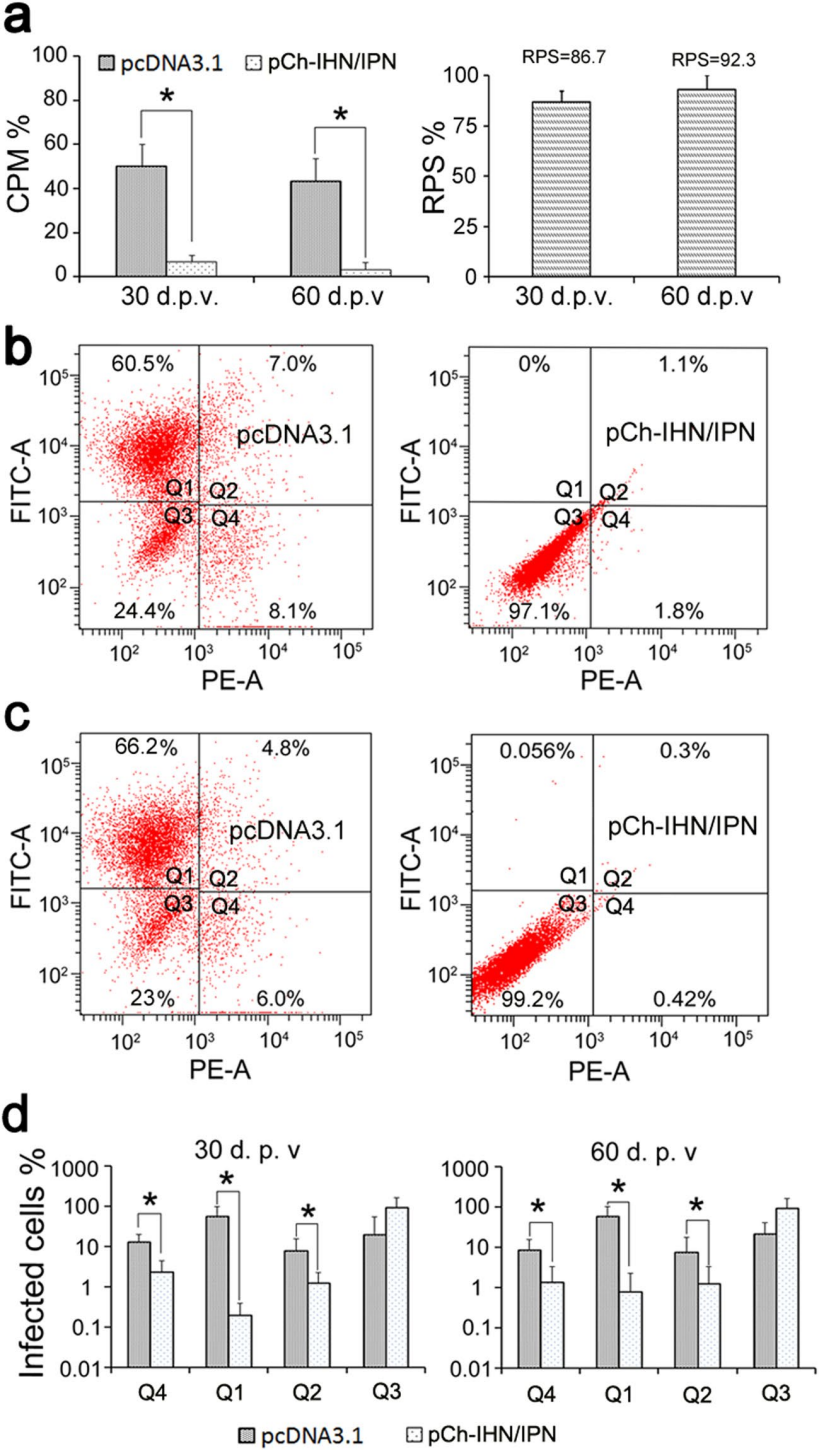
Evaluation of the protection afforded by the bivalent DNA vaccine against co-infection with IHNV and IPNV. Cumulative percentage mortality curves (**a**) and flow-cytometric quantification of IHNV and IPNV in the tissues of rainbow trout (n = 5) challenged with IHNV and IPNV at 30 and 60 d.p.v. (**b**,**c**) Dual protection afforded by the vaccine against co-infection. Q1: IPNV-infected cells; Q2: dual-infected cells; Q3: uninfected cells; Q4: IHNV-infected cells. The average proportions of virus-infected cells are shown in the histograms (**d**). Rainbow trout injected with plasmid pcDNA3.1 were used as the negative controls. *Significantly different (P < 0.05).

Replicate groups of 30 rainbow trout fry (5 g), injected i.m. with 1.0 µg of pCh-IHN/IPN or treated with the various controls, were challenged with a mixture of IHNV sn1203 and IPNV ChRtm213 at 30 or 60 d.p.v. In this experiment, the pCh-IHN/IPN-vaccinated rainbow trout challenged at 30 and 60 d.p.v. were protected significantly better than the pcDNA3.1-vaccinated fish (P < 0.05), with 3.33–6.67% cumulative mortality compared with 43.3–50.0% in the pcDNA3.1-mock-vaccinated groups. There was no significant difference in RPS at 30 and 60 d.p.v. (Fig. 4a; P > 0.05). There was no cross-reaction between IHNV neutralizing antibodies (NAbs) and IPNV or between IPNV NAbs and IHNV (see Supplementary Figure S3 for cross-reaction tests). Viruses were recovered at 15 days post-challenge from the tissue pools from the challenged trout in Chinook salmon embryo (CHSE-214) cells, analysed with an IFAT, and quantified with flow cytometry. The proportions of IHNV-infected cells and IPNV-infected cells were 1.8% and 0%, respectively (challenged at 30 d.p.v.) and 0.42% and 0.056%, respectively (challenged at 60 d.p.v.) in cells inoculated with tissues from pCh-IHN/IPN-vaccinated trout. However, they were 8.1% and 60.5%, respectively (30 d.p.v.) and 6.0% and 66.2%, respectively (60 d.p.v.) in cells inoculated with tissues from pcDNA3.1-mock-vaccinated trout (Fig. 4b,c). Thus, significantly fewer IHNV and IPNV were detected in the CHSE-214 cells inoculated with tissues from the pCh-IHN/IPN-vaccinated trout than in cells inoculated with tissues from the pcDNA3.1-mock-vaccinated trout (Fig. 4d; P < 0.05).

### Gene expression

**Figure 5:**
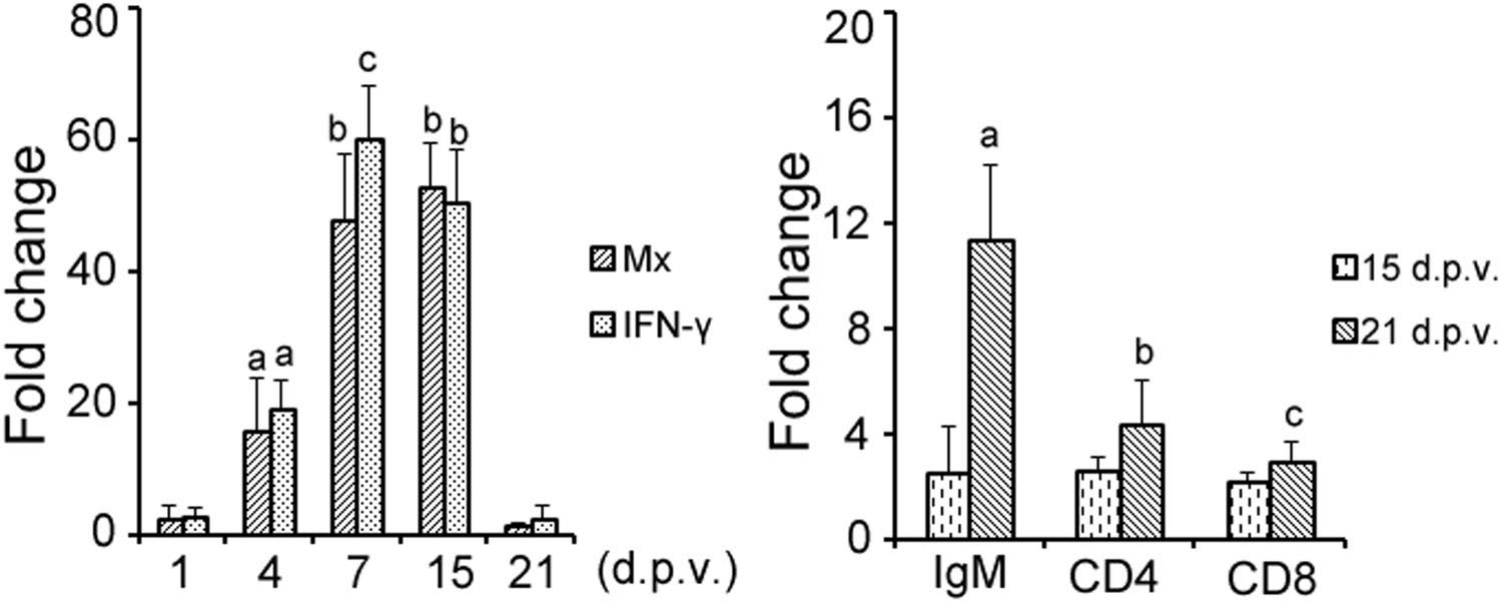
Fold changes in the expression of immune-related genes induced by the combined DNA vaccine in rainbow trout (n = 5). β-Actin was used to normalize the transcription of each gene in anterior kidney samples from rainbow trout at 1, 4, 7, 15, and 21 days post-vaccination (d.p.v.). The fold changes in their expression were calculated relative to their expression in the pcDNA3.1-vaccinated group. The differences were analysed, and different symbols above the bars indicate significant differences (P < 0.05).

The expression levels of Mx-1 and interferon γ (IFN-γ) in the anterior kidneys of the vaccinated fish were determined at 1, 4, 7, 15, and 21 d.p.v., and those of IgM, CD4, and CD8 were assessed at 15 and 21 d.p.v. The fold changes in expression were calculated relative to the levels in the pcDNA3.1-mock-vaccinated groups. The expression of the Mx-1 and IFN-γ genes was significantly upregulated in the pCh-IHN/IPN-treated trout at 4, 7, and 15 d.p.v. (Fig. 5; P < 0.05). The highest fold changes in Mx-1 and IFN-γ were 53-fold and 60-fold, respectively, which were observed at 15 and 7 d.p.v., respectively (Fig. 5). These results indicate that the bivalent DNA vaccine induced nonspecific immune responses in the rainbow trout as early as 4 d.p.v., which lasted until 15 d.p.v. The IgM expression detected in the kidneys at 15 d.p.v. increased significantly at 21 d.p.v (around 12-fold; P < 0.05). The expression levels of CD4 and CD8 were not as high as those of IgM, but significant changes were still observed at 21 d.p.v. (Fig. 5; P < 0.05).

### Neutralizing antibodies

Serum samples from vaccinated fish (n = 10) that had not been challenged with either virus were tested for seroconversion at 30 and 60 d.p.v. IHNV NAbs and IPNV NAbs were detected in all the serum samples. Most pCh-IHN/IPN-vaccinated fish had high titres (≥160) of both IHNV NAbs and IPNV NAbs (Table 1), whereas no NAbs were detected in the fish injected with the pcDNA3.1 vector (not shown). Positive control sera used in this study had titres >160 in all assays. The half-maximal inhibitory concentrations (IC_50_) of IHNV NAbs and IPNV NAbs were calculated as fold dilutions of serum. The IC_50_ of IHNV NAbs and IPNV NAbs were around 133 and 135, respectively, at 30 d.p.v, and 140 and 157 respectively, at 60 d.p.v. For IPNV, IC_50_ was significantly higher at 60 d.p.v. than at 30 d.p.v. (Table 1; P < 0.05). These results indicated that the bivalent DNA vaccine induced specific immune responses in the rainbow trout as early as 30 d.p.v., which persisted until 60 d.p.v.

**Table 1:** Neutralizing antibodies (NAb) induced by pCh-IHN/IPN in rainbow trout.

Test time (d.p.v.)	Viruses tested	Number of fish seroconverted/number tested	Nab titres (number of individual fish at each titre)	IC_50_
30	IHNV Sn1203	10/10	80 (3), ≥160 (7)	133.1 ± 3.56^a^
	IPNV ChRtm213	10/10	40 (1), 80 (1), ≥160 (8)	135.1 ± 0.31^a^
60	IHNV Sn1203	10/10	40 (1), 80 (1), ≥160 (8)	140.5 ± 0.70^b^
	IPNV ChRtm213	10/10	80 (1), ≥160 (9)	157.3 ± 4.3^c^

Differences were analysed, and different letters indicate significant differences (P < 0.05).

**Figure 6:**
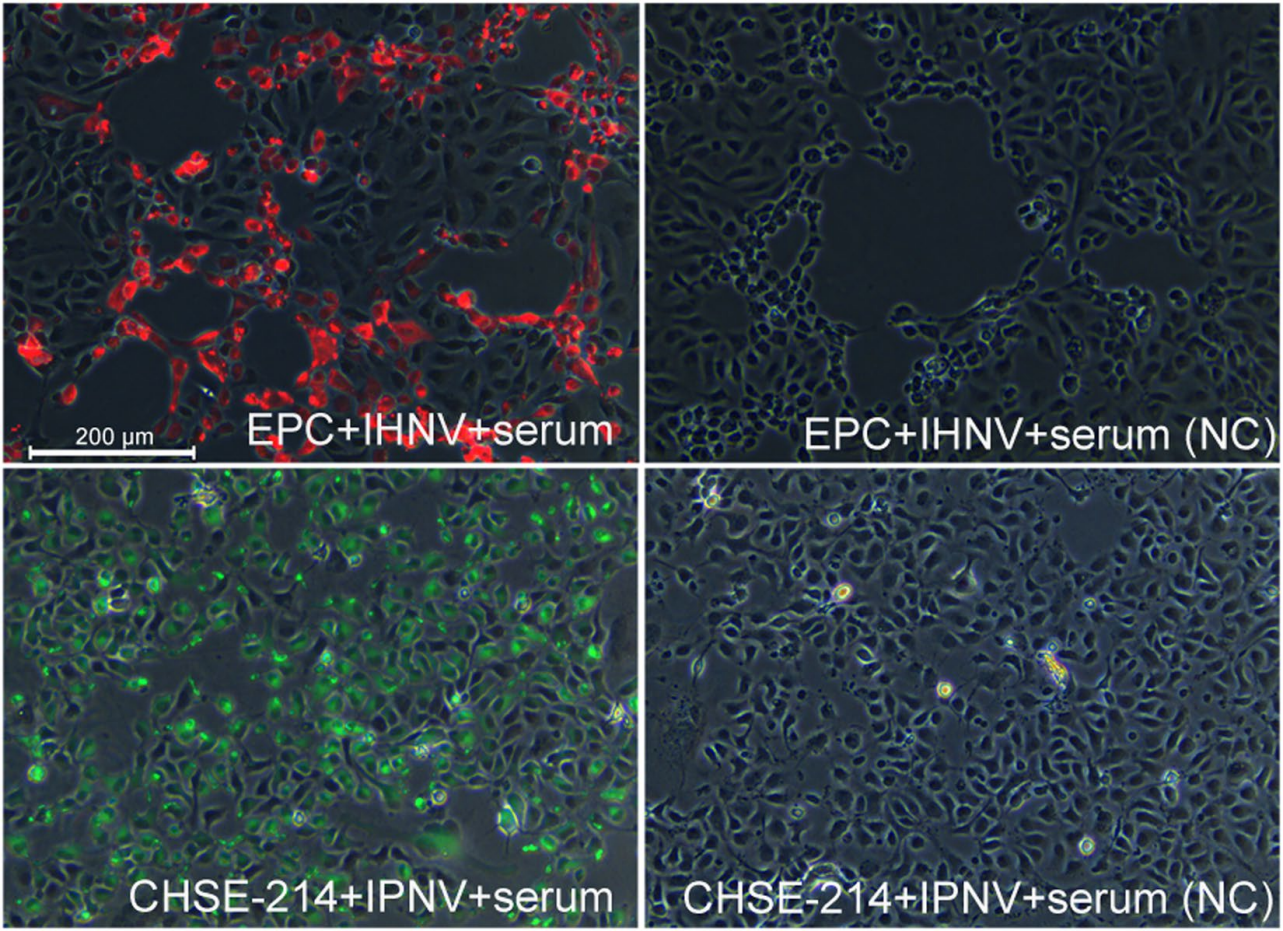
Specificity of NAb serum for IHNV and IPNV. Virus-infected cells were incubated with NAb-containing serum (from vaccinated trout), a rabbit polyclonal antibody directed against rainbow trout IgM Fc, and a fluorescently labelled secondary antibody. Sera from trout vaccinated with pcDNA3.1 were used as the negative controls. NC: negative control.

To determine the specificity of the antibodies elicited against IHNV and IPNV, a serum pool was used as the first antibody in an IFAT. Specific red and green fluorescence was observed in virally infected cells incubated with serum extracted from the pCh-IHN/IPN-vaccinated trout, whereas no specific fluorescent signal was observed in the virally infected cells incubated with serum extracted from the pcDNA3.1-vaccinated trout (Fig. 6).

## Discussion

The first outbreaks of IHN and IPN were recorded in juvenile fish in rainbow trout hatcheries in northeast China in 1985 and 1986^34^, respectively. The IHNV and IPNV strains have evolved in the decades since they were first introduced into China. The world’s IHNV isolates have been divided into five genogroups, M, L, U, E, and J, based on the glycoprotein gene sequence. All Chinese IHNV isolates belong to genogroup J, together with the other Asian IHNV strains^35, 36^. Although there is only one IHN viral serotype^37^, mutations in the G gene can cause low virulence or produce neutralization-resistant variant IHNV strains^38^. Previous IHNV vaccines have been generated with genogroup M and U IHNV isolates^13, 37, 39^. The worldwide IPNV isolates consist of nine serotypes^33^, and IPNV vaccines have been developed with the Spanish IPNV strain Sp^5, 22, 24, 40^, the Norwegian strain NVI015^41^, and the American VR299 strain^16^. There is no report of a vaccine developed from a Chinese IHNV or IPNV strain. IHN and IPN remain the most important diseases threatening salmonid aquaculture in China. Therefore, in this study, a bivalent DNA vaccine was produced from Chinese IHNV and IPNV strains. In this bivalent DNA vaccine, the IHNV G gene was inserted downstream from the *Cytomegalovirus* (CMV) promoter. The IPNV VP2 and VP3 genes were linked by a sequence encoding a flexible linker peptide (Gly_4_Ser)_3_ and inserted downstream from the internal ribosome entry site (IRES), which is responsible for expressing the VP2–VP3 genes. The bivalent DNA vaccine provided robust protection against Chinese IHNV and IPNV and against co-infection with the two viruses.

Previous studies have shown that IHNV DNA vaccines delivered by i.m. injection are safe, stable, and quickly produced, and induce robust nonspecific and specific immune responses^42^. A single nanogram dose provided almost complete protection to rainbow trout (3 g)^37^. In this study, we did not test the protection efficacy of lower doses. In a previous study^43^, the minimal dose of 1 μg of an IHNV DNA vaccine was determined and used throughout that study, and the bivalent DNA vaccine in the present study was constructed based on that IHNV DNA vaccine. Therefore, we used a single dose of 1.0 µg to vaccinate the rainbow trout (5 g). This dose is far lower than that used in mammals^37, 44, 45^. Fortunately, high protective efficacy conferred by the bivalent DNA vaccine was obtained at this dose. Protection as strong as that conferred by a DNA vaccine was also conferred by an attenuated IHNV vaccine delivered by nasal immunization. However, the attenuated vaccine was virulent and caused a low rate of mortality in the vaccinated trout^39^. Therefore, the IHNV DNA vaccine was safer than the attenuated IHNV vaccine. Some progress has been made in developing oral IHNV vaccines, an oral DNA vaccine^46^ and an oral yeast-surface-displayed vaccine^47^, which provide significant protection to trout. However, their protective efficacy was not as high as that of a DNA vaccine delivered by injection. These findings indicate that although the injection protocol is labour-intensive and time-consuming, an IHNV DNA vaccine delivered by i.m. injection is very safe and highly efficient.

Most vaccine efficacy tests determine the post-challenge RPS as a measure of protection. However, no mortality was detected in our IPNV challenge experiments, so the IPNV load or IPNV NAb titre was measured in the vaccinated trout as an indicator of protective efficacy, as described in previous studies^9, 27, 40^. In this study, both the IPNV load and IPNV NAb titre were determined. The IPNV load decreased dramatically and a high IPNV NAb titre was generated in the bivalent-DNA-vaccine-treated trout, indicating that good protection against IPNV infection was afforded by the bivalent DNA vaccine. The structural protein(s) of many viruses form specific aggregates through self-assembly in a variety of different expression systems^48^. These virally derived particles generally imitate the native viruses in size and morphology, and are referred to as ‘virus-like particles’ (VLPs)^27^. When viral proteins aggregate to form particles that do not mimic the viral capsid in size, but still form predictable complex(es), they are referred to as ‘subviral particles’ (SVPs). In previous studies, VLPs^26^ and SVPs^27^ against IPNV were designed with the VP2–VP3 or VP2 genes alone, and their protective efficacy was confirmed with IPNV NAb titration or by measuring the IPNV load. In our study, VP2–VP3 expressed by the bivalent DNA vaccine vector induced specific immune responses and reduced the IPNV load in the vaccinated rainbow trout, indicating that the VP2–VP3 protein linked by (Gly_4_Ser)_3_ was efficiently expressed and correctly folded. We did not determine whether VP2–VP3 was assembled into VLPs in this study. Since VLPs assemble in a variety of different expression systems, we speculate that the VP2–VP3 probably assembles into VLPs in fish cells.

Previous studies have reported that interactions between IHNV and IPNV can cause a loss of infectivity and reduce the infective titre^49, 50^. In the present study, the cumulative percentage mortality (CPM) of the mock-vaccinated rainbow trout co-infected with IHNV and IPNV was significantly lower than that of fish challenged with IHNV alone, which is consistent with previous results. In addition to the measurement of CPM, the viruses were recovered from the vaccinated trout and quantified with flow cytometry. CPM and viral quantification both showed that the pCh-IHN/IPN-vaccinated rainbow trout were significantly protected from co-infection, and almost no IHNV or IPNV was recovered from them. However, significantly high levels of IHNV and IPNV were recovered from the pcDNA3.1-mock-vaccinated rainbow trout, confirming that the bivalent DNA vaccine protected trout from co-infection by IHNV and IPNV.

Farmed fish are susceptible to different infectious disease agents, including viruses and bacteria. In a previous study, rainbow trout were doubly nasally vaccinated with an attenuated IHNV vaccine and a formalin-killed enteric red mouth bacterium^29^. The authors reported that dual vaccination against two different pathogens *via* the nasal route is a very effective vaccination strategy in aquaculture, particularly when the two vaccines are introduced separately into different nares, although this makes dual vaccination more complex than single vaccination. Therefore, multivalent vaccines against two or more pathogens are more practical when vaccinating cultured animals. The bivalent DNA vaccine produced in this study contained the two antigen genes in one vector. The vaccine did not require premixing before vaccination, and a single injection of the vaccine protected the rainbow trout from attack by both viruses. Although the injection protocol is labour-intensive and time-consuming, the fact that a single injection can provide protection against two acute viral pathogens is very attractive. Therefore, the bivalent DNA vaccine should play an important role in the control of IHN and IPN in China and inspire more strategies for controlling two or more pathogens with one vaccine.

## Methods

### Ethics statement

In this study, all animal experiments strictly followed protocols approved by the Animal Welfare Committee of China Agricultural University (permit number: XK662) and the study was carried out in strict accordance with the guidelines and regulations established by this committee.

### Fish, viral strains, and cell lines

Specific-pathogen-free rainbow trout (mean weight, 5 g) were maintained in 50 L tanks with circulating water at 15 °C and fed a dry pelleted diet *ad libitum*. The Ja serotype of IPNV ChRtm213^33^ and the J genotype IHNV Sn1203^32^ are laboratory stocks. IPNV ChRtm213 was propagated in CHSE-214 cells and IHNV Sn1203 was propagated in EPC cells, as described previously^16, 51^. The EPC cell line was originally deposited in the American Type Culture Collection (ATCC) as a carp (*Cyprinus carpio*) cell line, but has since been identified as derived from fathead minnow (*Pimephales promelas*; ATCC CRL-2872). It has also been used as a cell line for plasmid transfection because of its high transfection efficiency^20^.

### Constructing the bivalent DNA vaccine

The G gene of IHNV Sn1203 was cloned into the multiple cloning site of the pcDNA3.1 vector using *Bam*H I and *Not* I, to construct the recombinant plasmid pcDNA-IHN. The VP2 and VP3 genes of IPNV ChRtm213 were linked to a sequence encoding a (Gly_4_Ser)_3_ linker^52^ (Xu *et al*., 2014a) with overlapping PCR, to create the fused VP2–VP3 genes. The VP2–VP3 genes were cloned downstream from the IRES in pT-IRES with *Sal* I and *Xho* I, to construct the fused IRES–VP2–VP3 genes. The IRES–VP2–VP3 genes were cloned downstream from the G gene in the pcDNA-IHN vector with *Not* I and *Xho* I, to construct the bivalent DNA vaccine pCh-IHN/IPN. The bivalent DNA vaccine pCh-IHN/IPN and pcDNA3.1 were prepared with a plasmid extraction kit (Tiangen, Shanghai, China). A map of the recombinant plasmid pCh-IHN/IPN is given in Supplementary Figure S1 with the SnapGene Viewer software.

### *In vitro* expression of antigen genes

The *in vitro* expression of the G and VP2–VP3 genes from the bivalent DNA vaccine in fish cells was confirmed by the transfection of EPC cells with the vaccine, followed by an IFAT, as described previously^6^. A rabbit polyclonal antibody directed against IHNV glycoprotein^53^ and a Cy3-conjugated goat anti-rabbit-IgG secondary antibody (cat. no. ab97075; Abcam, Cambridge, England) were used to determine the expression of the G protein, and a mouse anti-IPNV-VP3 polyclonal antibody (prepared with routine procedures) and a FITC-conjugated goat anti-mouse-IgG secondary antibody (cat. no. ab6785; Abcam) were used to detect the expression of the VP2–VP3 fusion protein.

### *In vivo* expression of antigen genes

A dose of 1.0 µg of pCh-IHN/IPN was used in all vaccination experiments. Rainbow trout were anesthetized by immersion in tricaine methane sulfonate (MS-222; Sigma, St. Louis, MO, USA), and the bivalent DNA vaccine was delivered by i.m. injection at the base of the dorsal fin. Rainbow trout injected with the empty pcDNA3.1 vector were used as the negative control. The *in vivo* expression of the G and VP2–VP3 proteins was confirmed with the western blotting of muscle samples from rainbow trout (n = 5) vaccinated with pCh-IHN/IPN or pcDNA3.1, collected 3, 7, and 15 d.p.v. β-Actin was used as the reference protein. Western blotting was performed as described previously^54^, and the expression of the G protein was visualized with a rabbit anti-IHNV-glycoprotein polyclonal antibody and a horseradish peroxidase (HRP)-conjugated goat anti-rabbit-IgG secondary antibody (cat. no. sc-2004; Santa Cruz Biotechnology, Inc., Santa Cruz, CA, USA). The expression of the VP2–VP3 protein was visualized with a mouse anti-IPNV-VP3 polyclonal antibody and an HRP-conjugated goat anti-mouse-IgG secondary antibody (cat. no. sc-2005; Santa Cruz Biotechnology).

### Challenge with IHNV and CPM

Rainbow trout vaccinated with pCh-IHN/IPN or empty pcDNA3.1 were challenged with IHNV Sn1203 at 30 and 60 d.p.v. Duplicate groups of 30 fish were anesthetized and injected i.p. with 10^2^ plaque-forming units (PFU) of IHNV Sn1203 in 100 µl of phosphate-buffered saline (PBS). Mock infections were performed by replacing the viral suspension with PBS. CPM was recorded daily in parallel experiments for 21 days. RPS was then calculated with the formula: RPS = [1 − (% mortality of fish given vaccine/% mortality of fish given pcDNA3.1)] × 100^22^.

### Challenge with IPNV and IPNV load

Duplicate groups of 50 rainbow trout vaccinated with pCh-IHN/IPN or pcDNA3.1 were injected i.p. with IPNV ChRtm213 at a dose of 10^6^ PFU in 100 µl of PBS at 30 or 60 d.p.v. Trout vaccinated with the pcDNA3.1 vector alone were challenged and used as the negative control. The anterior kidneys were collected from the rainbow trout (n = 5) vaccinated with pCh-IHN/IPN or empty pcDNA3.1 at 15 days after challenge to evaluate the effect of the vaccine on viral clearance and the viral load^20, 27^. RNA was extracted from the individual samples with TRIzol Reagent (cat. no. 15596-018; Invitrogen, CA, USA). IPNV VP1 gene expression was determined with quantitative reverse transcription–PCR (qRT–PCR) using the One Step SYBR PrimeScript PLUS RT–PCR Kit (Perfect Real Time) (cat. no. RR096A; Takara, Shiga, Japan) and previously published primers^55^. EF1-α was used as the reference gene against which the expression of the IPNV VP1 gene was normalized^27^.

### Simultaneous challenge with IHNV and IPNV

To determine the protection afforded by the bivalent DNA vaccine against co-infection by IHNV and IPNV, rainbow trout vaccinated with pCh-IHN/IPN or empty pcDNA3.1 were challenged with an i.p. injection of a mixture of 10^2^ PFU of IHNV Sn1203 and 10^6^ PFU of IPNV ChRtm213 in 100 µl of PBS at 30 or 60 d.p.v. CPM was recorded daily for 21 days in parallel experiments.

Fish (n = 5) that survived for 15 days after challenge (when mortality ceased) were sampled. Their liver, spleen, and anterior kidney tissues were removed and pooled, and the virus was propagated in CHSE-214 cells. When subtle cytopathic effects were detected, the CHSE-214 cells were incubated with primary and secondary antibodies, as described for the IFAT in this study. The CHSE-214 cultures were then digested with 0.25% trypsin, and the IHNV and IPNV antigens in the CHSE-214 cells were quantified with a FACSAria™ Cell Sorter (BD Biosciences, San Jose, CA, USA).

### Gene expression

The expression levels of IFN-γ and Mx-1, markers of the nonspecific innate immune response to viruses^56^, were measured at 1, 4, 7, 15, and 21 d.p.v. to assess the nonspecific immune response induced by the bivalent vaccine. On days 15 and 21 post-vaccination, the expression of IgM^57^ was also determined to evaluate the adaptive immune response. The transcription of CD4 and CD8 Th-cell markers was also assessed^17^. The RNA from the anterior kidney samples (n = 5) was prepared with TRIzol Reagent. qRT–PCR was performed with the One Step SYBR PrimeScript PLUS RT–PCR Kit (Perfect Real Time; Takara). The β-actin gene was used as the reference gene against which the gene expression levels were normalized^58^. The fold changes in gene expression were calculated relative to their expression in the mock-vaccinated control group (treated with pcDNA3.1 in PBS).

### Characterization and titration of NAbs

Blood samples were collected by caudal transection from rainbow trout (n = 10) vaccinated with pCh-IHN/IPN or pcDNA 3.1 at 30 or 60 d.p.v., and the sera were prepared with a routine procedure^59^. The NAb titre of each serum sample was determined with a complement-dependent neutralization assay. Titres ≥20 were considered positive and titres <20 were considered negative^60^. The IC_50_ values of the pooled sera were measured with a routine procedure and calculated with the software GraphPad Prism 5. Serum samples were also used as the first antibody in an IFAT to characterize their specificity for IHNV and IPNV. The IFAT was performed as described in a previous study^6^, and a rabbit polyclonal antibody directed against rainbow trout IgM Fc^61^ was used to link the first antibody in the serum to fluorescently labelled goat anti-rabbit antibodies. EPC cells were used for the titration of IHNV NAbs, and CHSE-214 cells were used for the titration of IPNV NAbs. Serum samples from trout treated with pcDNA3.1 were used as the negative control.

### Statistical analysis

Analysis of variance was used to assess the differences in gene expression levels. Student’s *t* test was used to compare some paired samples. P < 0.05 was considered significant.

## Electronic supplementary material

Supplementary Information
