# Supplementary material for: Bivalent DNA vaccine induces significant immune responses against infectious hematopoietic necrosis virus and infectious pancreatic necrosis virus in rainbow trout
Source: Sci Rep. 2017 Jul 18;7:5700. doi: 10.1038/s41598-017-06143-w (PMC5515949; doi:10.1038/s41598-017-06143-w)
Supplement: Supplementary file 1 — Supplementary Information [file 41598_2017_6143_MOESM1_ESM.doc]

# *Nature-Scientific Reports*

## Supplementary materials

### Bivalent DNA vaccine induces significant immune responses against infectious hematopoietic necrosis virus and infectious pancreatic necrosis virus in rainbow trout

Liming Xu^1^, Jingzhuang Zhao^1^, Miao Liu^1^, Guangming Ren^1^, Feng Jian^2^, Jiasheng Yin^1^, Ji Feng^1^, Hongbai Liu^1^, and Tongyan Lu^1^ *

^1^Heilongjiang River Fishery Research Institute Chinese Academy of Fishery Sciences, Harbin 150070, PR China

^2^Benxi Agrimarine limited company, Benxi 117000, PR China

e-mail addresses:

LM, [279463437@qq.com](mailto:279463437@qq.com)

JZ, [422842454@qq.com](mailto:422842454@qq.com)

ML, [446048847@qq.com](mailto:446048847@qq.com)

GR, [171066690@qq.com](mailto:171066690@qq.com)

JF, jfeng666@126.com

JY, [xwsc20@tom.com](mailto:513055233@qq.com)

FJ, jifeng-hlj@163.com

HL, [112893129@qq.com](mailto:112893129@qq.com)

TL, [lutongyan@hrfri.ac.cn](mailto:lutongyan@hrfri.ac.cn)

*Corresponding author:

Tong-Yan Lu

Heilongjiang River Fishery Research Institute Chinese Academy of Fishery Sciences, Harbin 150070, PR China

Tel/Fax: +86 451 84869341

E-mail: lutongyan@hrfri.ac.cn


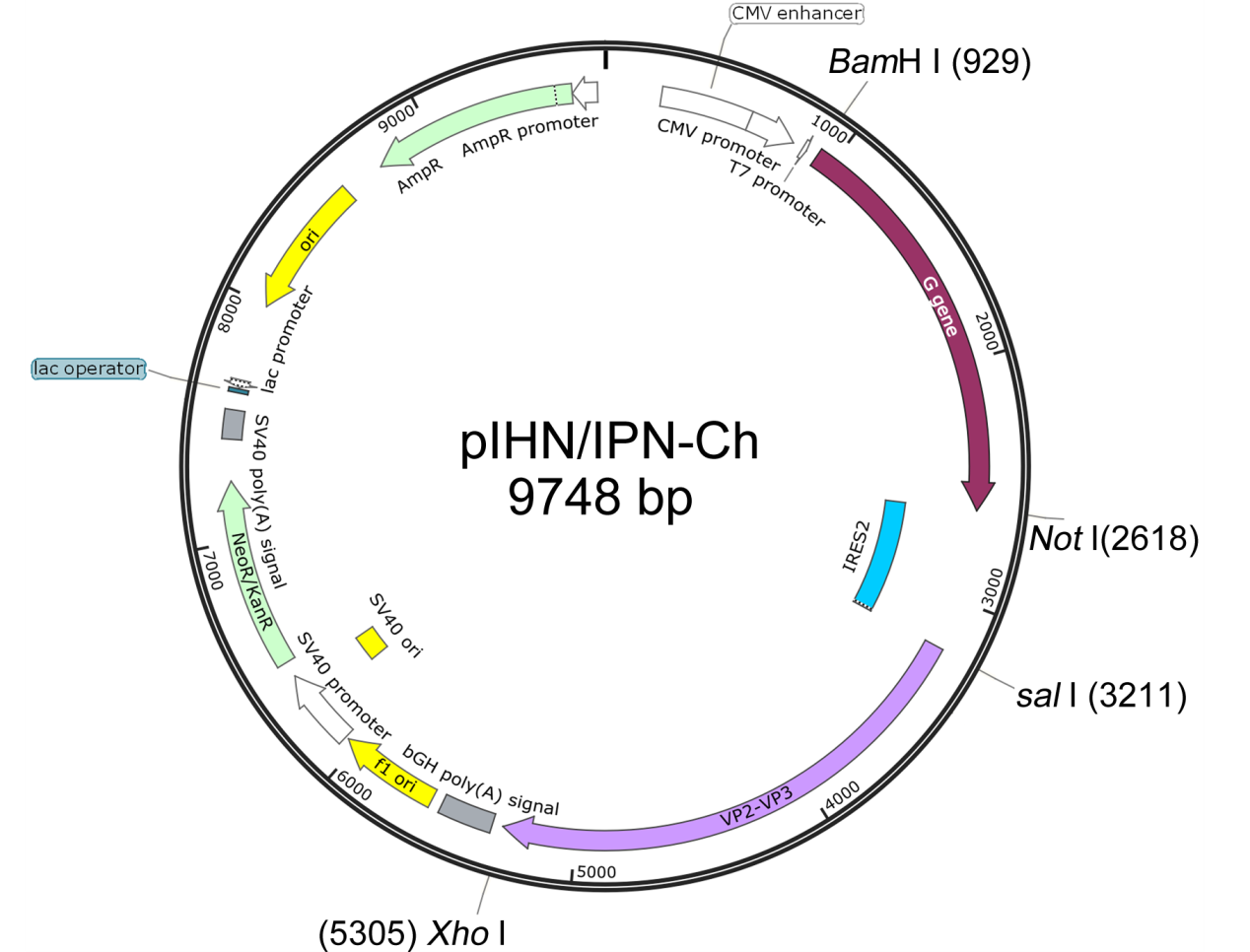


**Figure S1.** Schematic map of the bivalent DNA vaccine pCh-IHN/IPN. G: the glycoprotein of the Chinese infectious hematopoietic necrosis virus strain Sn1203 (IHNV); VP2-VP3: the VP2-VP3 fusion gene of the infectious pancreatic necrosis strain ChRtm213; IRES: internal ribosome entry site.


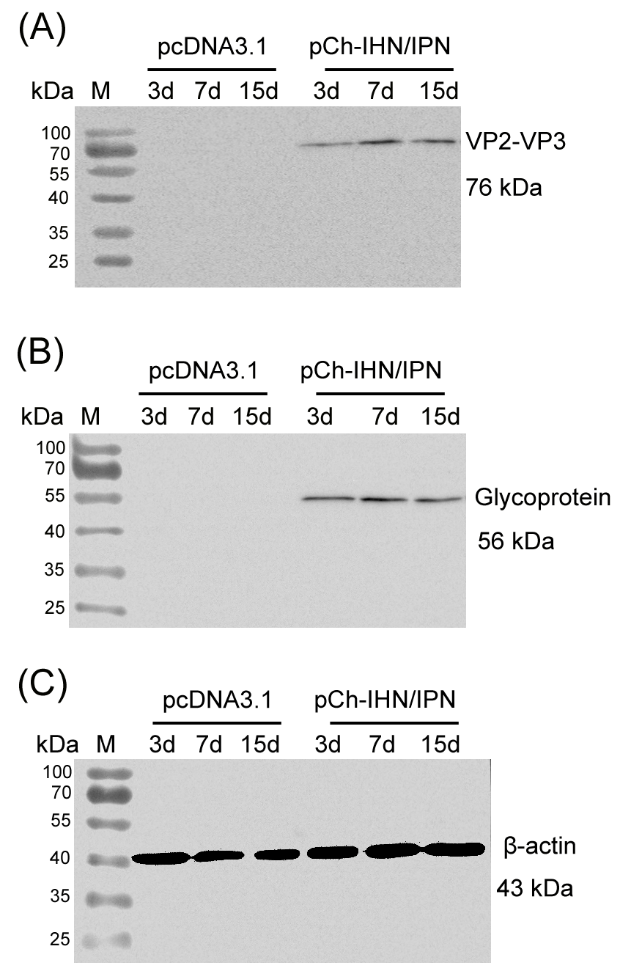


**Figure S2.** Full length gels of Western blotting of muscle samples from vaccinated rainbow trout (n = 5), collected at 3, 7, and 15 days post-vaccination.

**
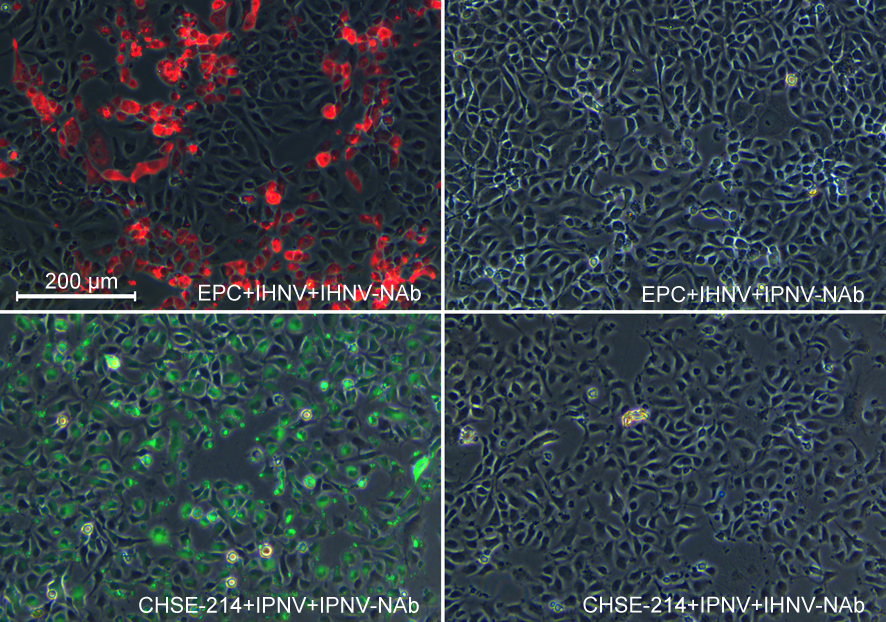
**

**Figure S3.** Cross binding of viral neutralizing antibodies (NAbs) to IHNV and IPNV. Virus-infected cells were incubated with viral NAb-containing serum, a rabbit polyclonal antibody directed against rainbow trout IgM Fc, and a fluorescently labelled secondary antibody.
